# Tuberculous cold abscess of sternoclavicular joint: a case report

**DOI:** 10.11604/pamj.2020.36.16.20697

**Published:** 2020-05-12

**Authors:** Ismail Burud, Mohammad Arshad Ikram, Mahadevan Deva Tata, Junalina Jaafar

**Affiliations:** 1 Department of Surgery, International Medical University, Clinical Campus, Seremban, Malaysia; 2 Orthopaedic Department, International Medical University, Clinical Campus, Seremban, Malaysia; 3 Department of Surgery, Hospital Tuanku Jaafar, Seremban, Malaysia; 4 Department of Histopathology, Hospital Tuanku Jaafar, Seremban, Malaysia

**Keywords:** Swelling of sternoclavicular joint, cold abscess, tuberculosis, extrapulmonary tuberculosis

## Abstract

Bone and joint tuberculosis is a serious medical problem; tuberculosis of sternoclavicular joint is rare. We present a case of a healthy 37-year old man with sternoclavicular joint tuberculosis. The subject presented with a three weeks history of left sternoclavicular joint painless swelling without fever or weight loss. He had no previous history of pulmonary tuberculosis. Laboratory testing revealed erythrocyte sedimentation rate of 70 mm/hour, C-reactive protein of 30 mg/liter and a normal leucocyte count. Biopsy of the lesion showed caseous necrosis and pus culture revealed *Mycobacterium tuberculosis*. He was treated with joint debridement and anti-tuberculous medications. Tuberculosis resolved completely but post-infection patients had residual joint arthritis. Tuberculosis may infect unusual joints such as the sternoclavicular joint.

## Introduction

Tuberculosis (TB) is still a very common disease in developing countries. TB arthritis is seen among children and young adults in endemic regions of the developing countries whereas in the developed countries it is seen in the older population and immunocompromised patients. It is a chronic disease that may affect any part of the human body. TB of the sternoclavicular joint is extraordinarily rare and can raise diagnostic problems. Osteoarticular lesions usually occur following paucibacillary haematological dissemination by fixation of a colony inside the active bone marrow. Tuberculous etiology should be considered for patients presenting with atypical sites of skeletal inflammation. A high degree of clinical suspicion and familiarity with the various radiologic manifestations of tuberculosis and sending specimens for mycobacterial culture allow early diagnosis and timely initiation of appropriate therapy, thereby reducing patient morbidity [1]. We describe a case of an adult who had a tuberculous infection in the sternoclavicular joint and the surrounding tissues. Computerized tomography greatly enhanced the understanding of the extent of the disease process. Tuberculosis (TB) is still a very common disease in developing countries. TB arthritis is seen among children and young adults in endemic regions.

## Patient and observation

**Figure 1:**
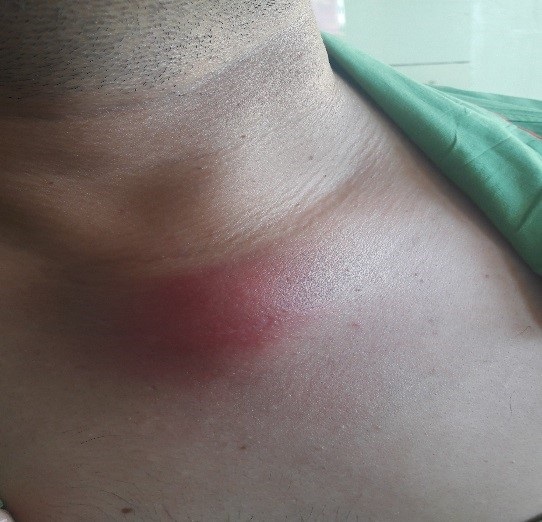
Swelling of the left sternoclavicular joint

**Figure 2:**
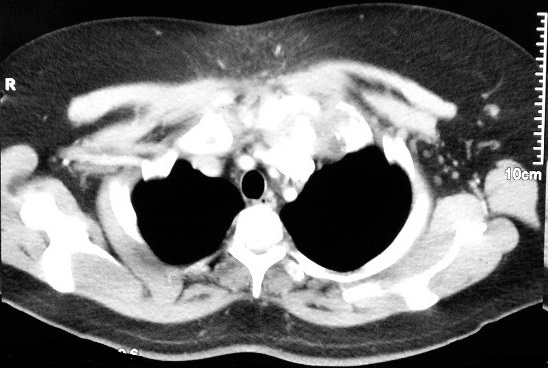
CT scan showing lobulated collection in the subcutaneous tissue of the left lower neck extending into left sternoclavicular and first sternochondral joint

**Figure 3:**
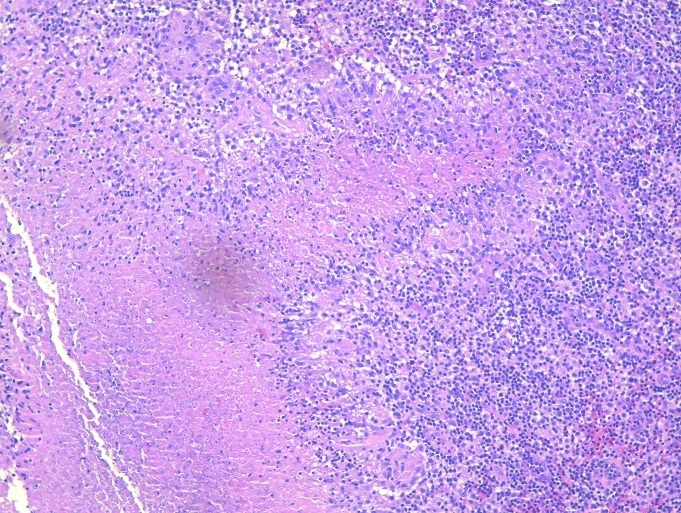
Granulomatous lesion with caseating necrosis palisaded by epithelioid histiocytes, lymphocytes and occasional Langhan´s type giant cells

**Figure 4:**
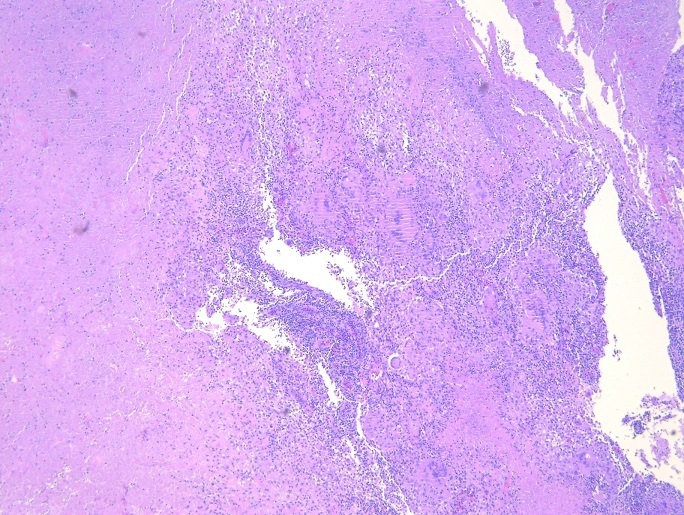
Multiple granulomatous lesion with large caseous necrosis

A healthy 37-year-old man presented with complaints of swelling and pain involving the left sternoclavicular joint region of three weeks´ duration in September 2017. There was no history of fever, loss of appetite or loss of weight. He was treated with antibiotics for one week by his family doctor. The patient had no history of drug abuse or high-risk behavior. He has primary infertility for which he was investigated and received hormonal therapy. There was no family history of tuberculosis. On general physical examination, his vital signs were normal. Body mass index was 34 kg/m^2^. No cervical or axillary lymphadenopathy. There was a swelling measuring 4x4 cm over the left sternoclavicular joint region. The skin over the swelling was stretched and erythematous (Figure 1). It was non-tender and soft inconsistency. Movements of the left shoulder joint were normal. The chest was clear; heart sounds were normal. Abdominal examination was normal. Full blood count revealed haemoglobin of 11.8 g/dl, white cell count of 6.8x10^3^ μl, neutrophil 57.7%, lymphocytes 33.4%, eosinophil 2.6%, monocyte 5.7%, basophil 0.6%. The erythrocyte sedimentation rate (ESR) was elevated to 70 mm/hour, and the test for C-reactive protein (CRP) was positive (thirty milligrams per liter). A serological test done for HIV was negative. A tuberculin skin test was also negative. Ziehl Neelson stain was negative. Chest radiograph revealed normal lung fields with suspicious of the irregularity of right sternoclavicular joint. A computerized tomographic scan showed extensive destruction involving cortices of clavicular as well as the sternal end of the joint with loculated collection in the subcutaneous tissue at the left side of lower neck extending into the left sternoclavicular and first sternochondral joint with the impression of septic arthritis in these two joints (Figure 2). Ceftriaxone one gram intravenously was commenced. An open biopsy was performed. Frank yellow creamy pus found in the subcutaneous communicating with the joint. Culture of pus identified as *Mycobacterium tuberculosis*. Biopsy of the abscess wall showed multiple granulomatous lesion with large caseous necrosis (Figure 3, Figure 4). A diagnosis of tuberculous arthritis was made, based on the presence of granulomatous lesion and mycobacteria in the histological material. A hospital stay of the patient was 10 days. lsoniazid (300 mg per day), rifampin (600 mg per day), ethambutol (1600 mg per day), pyrazinamide (2000 mg per day), pyridoxine (20 mg per day) were administered for two months and then isoniazid (300 mg per day), rifampin (600 mg per day) and pyridoxine (20 mg per day) were continued for seven months. At a follow-up examination ten months after treatment, the patient recovered well and there was no evidence of recurrence of the infection clinically and by laboratory investigations, but he did show mild pain due to osteoarthritis of the sternoclavicular joint.

## Discussion

Tuberculosis is a major cause of morbidity and mortality worldwide especially in Southeast Asia and posing threat owing to the population growth worldwide. In Malaysia, there has been an increase in the number of cases of tuberculosis (TB) in the past few years [2]. Sternoclavicular joint TB arthritis is rare, comprising only 1-2% of TB arthritis cases [3], but there are always residual sequelae of the disease which may be due to delay in the diagnosis [4]. Dhillon *et al.* have described two types of sternoclavicular joint TB arthritis: an aggressive course with constitutional symptoms of joint destruction and a less aggressive type without constitutional symptoms and with minimal joint destruction [3]. This difference may be due to differences in the virulence of the organisms and host resistance. Sternoclavicular joint TB usually has a less aggressive course with swelling of the joint and minimal pain unlike that seen with other types of septic arthritis [5]. Jain *et al.* reported 13 cases of tuberculosis of the sternoclavicular joint in which all had painful swelling and only one culture was positive for TB [6]. In our case, there was no pain which suggests that it was not a regular bacterial infection. In our case, ESR, and CRP were markedly elevated suggesting infection. It is uncommon for a healthy patient to have extrapulmonary tuberculosis (EPTB). TB arthritis is often secondary to the common reason for tuberculosis arthritis and is secondary to either new or reactivated pulmonary TB [7]. Patients may have EPTB without pulmonary TB such as a case of sternoclavicular joint TB reported by Sahu, [1], and a case of TB pyomyositis of arm reported by Momin *et al*. [8]. A sternoclavicular joint TB is difficult to diagnose which may result in a delayed diagnosis and treatment. Conventional radiography is often not helpful for diagnosing sternoclavicular joint TB but computed tomography (CT) scan may be more useful. CT scan finding of sternoclavicular joint TB has been described by Shah *et al*. [5], which are bone and cartilage destruction, soft tissue masses crossing fascial planes with rim and diffuse enhancement (granulation tissue), calcifications and underlying pleuro-parenchymal tubercular. Surgical debridement at the time of open biopsy can promote healing [9]. A swollen sternoclavicular joint in a patient with a history of TB should raise the suspicion of sternoclavicular joint TB. But in our case due to the absence of the previous history of tuberculosis, constitutional symptoms and normal laboratory parameters, diagnosis becomes difficult. We agreed with Yasuda *et al*. [7], that advanced tuberculous arthritis and osteomyelitis in the sternoclavicular joint should be treated with a combination of thorough operative debridement and systemic administration of anti-tuberculous agents. This disease did not recur in our patient but Enarson *et al*. [3] reported that the risk for reactivation necessitates careful long-term follow-up. Based on our experience, sternoclavicular joint TB should be treated with a combination of surgical debridement and systemic anti-tuberculous medications.

## Conclusion

In conclusion, we report here a case of sternoclavicular joint TB successfully treated with surgical debridement and anti TB medications but resulted in destructive arthritis. Maintaining a high index of clinical suspicion and sending specimens for acid-fast bacillus (AFB) smear and mycobacterial culture reduce the chance of missing the diagnosis.

## Competing interests

The authors declare no competing interests.
